# Effects of physical activity on lung function and quality of life in asthmatic children: An updated systematic review and meta-analysis

**DOI:** 10.3389/fped.2023.1074429

**Published:** 2023-02-08

**Authors:** Zenghui Jing, Xingzhi Wang, Panpan Zhang, Jinli Huang, Yuanyuan Jia, Juan Zhang, Huajie Wu, Xin Sun

**Affiliations:** Department of Pediatrics, Xijing Hospital, The Fourth Military Medical University, Xi’an, China

**Keywords:** physical activity, lung function, quality of life, asthma, children

## Abstract

**Background and objectives:**

The benefits of physical activity (PA) for asthmatic children were increasingly recognized, and as the design of studies on PA and asthma has become more refined in recent years, the latest evidence needed to be updated. We performed this meta-analysis to synthesize the evidence available from the last 10 years to update the effects of PA in asthmatic children.

**Methods:**

A systematic search was conducted in three databases, PubMed, Web of Science, and Cochrane Library. Randomized controlled trials were included, and two reviewers independently conducted the inclusion screening, data extraction, and bias assessment.

**Results:**

A total of 9 studies were included in this review after 3,919 articles screened. PA significantly improved the forced vital capacity (FVC) (MD 7.62; 95% CI: 3.46 to 11.78; *p* < 0.001), and forced expiratory flow between 25% and 75% of forced vital capacity (FEF_25–75_) (MD 10.39; 95% CI: 2.96 to 17.82; *p* = 0.006) in lung function. There was no significant difference in forced expiratory volume in the first second (FEV_1_) (MD 3.17; 95% CI: −2.82 to 9.15; *p* = 0.30) and fractional exhaled nitric oxide (FeNO) (MD −1.74; 95% CI: −11.36 to 7.88; *p* = 0.72). Also, PA significantly improved the quality of life as assessed by the Pediatric Asthma Quality of Life Questionnaire (all items *p* < 0.05).

**Conclusions:**

This review suggested that PA could improve FVC, FEF_25–75_, and quality of life in asthmatic children, but there was insufficient evidence of improvement in FEV_1_ and airway inflammation.

**Systematic Review Registration:**

https://www.crd.york.ac.uk/PROSPERO/, identifier: CRD42022338984.

## 1. Introduction

Asthma is a heterogeneous disease characterized by chronic airway inflammation, and is the most common chronic respiratory disease in children. The Global Initiative for Asthma (GINA) highlights that the prevalence of asthma in children is increasing from year to year, particularly in developing countries and in young children among (1). The guidelines and consensus emphasize that the goals of treatment for asthmatic children are to control symptoms, reduce recurrence, and avoid exacerbations. Currently, the control treatment of asthma is still dominated by medications, including corticosteroids, bronchodilators and biologics. However, long-term use of medications inevitably has adverse effects on children's growth and development (2–4), especially with irregular asthma treatment, and also increases the economic burden. Therefore, scientists have begun to explore the positive role of non-pharmacological treatments in asthma management, including health education (5), exercise training (6), and pulmonary rehabilitation (7, 8), etc.

Exercise had been identified as a common trigger for asthma attacks or exacerbations in children (9, 10). Early on, during the treatment of children with asthma, physicians and caregivers overemphasized exercise-induced asthma and often reduced the duration and intensity of activity in asthmatic children. A recent study found that decreased aerobic fitness and increased sedentary time in children were associated with worsening asthma (11). As the link between exercise and asthma was studied in depth, some of the studies showed that regular exercise could improve the quality of life (QoL) and lung function in asthmatic patients (12, 13). However, the results of different reviews and meta-analyses often led to contradictory conclusions. For example, Joschtel B, et al. reported that exercise significantly improved cardiovascular fitness and QoL in asthmatic children (14), while another systematic review indicated there was insufficient evidence to identify the longitudinal effects of physical activity (PA) on lung function in children (15). Also, GINA only mentioned in its non-pharmacological strategies that regular PA could help to improve the management of asthma and promote children's participation in daily activities, but it remained uncertain what type or intensity of PA was beneficial for children in asthma.

Notably, an earlier meta-analysis summarized the effect of PA on asthmatic children, the authors also noted that the included studies were poorly designed. As a result, little progress was made on the relationship between lung function and PA in asthmatic children (16). Recently, some scholars wanted to study the effect of PA on asthma outcomes in adults, but no meta-analysis was performed due to the high heterogeneity of the included studies. And they concluded that PA improved lung function, QoL, and serum inflammatory markers in patients with asthma (17). Others conducted some meta-analyses of the effects of swimming on asthmatic children, but they did not consider other forms of exercise (18, 19). Therefore, we conducted this systematic review and meta-analysis focusing only on children and synthesized the evidence available for the last 10 years to update the effects of PA in asthmatic children.

## 2. Methods

This systematic review and meta-analysis was registered in the international prospective register of systematic reviews, and the registration number is CRD42020216469. Following the principles given in the Preferred Reporting Items for Systematic Reviews and Meta-analysis (PRISMA) statement, a search for randomized controlled trials (RCTs) investigating the effects of PA in asthmatic children was conducted (20).

### 2.1. Search strategy

The following databases were searched: PubMed, Web of Science and Cochrane Library, and all databases were limited to the search period 2010 to 2020. We described the search strategy of PubMed in Table 1. And we conducted the similar search strategy on the Web of Science and the Cochrane Library based on different specific requirements. Meanwhile, reference lists of relevant studies were also scanned.

**Table 1:** The search strategy in PubMed.

Search	Query
1	"asthma"[MeSH Terms] OR “asthma"[All Fields] OR “asthmas"[All Fields] OR “asthma s"[All Fields] OR (“asthma"[MeSH Terms] OR “asthma"[All Fields] OR (“bronchial"[All Fields] AND “asthma"[All Fields]) OR “bronchial asthma"[All Fields]) OR (“asthma"[MeSH Terms] OR “asthma"[All Fields] OR (“asthma"[All Fields] AND “bronchial"[All Fields]) OR “asthma bronchial"[All Fields])
2	"child"[MeSH Terms] OR “child"[All Fields] OR “children"[All Fields] OR “child s"[All Fields] OR “children s"[All Fields] OR “childrens"[All Fields] OR “childs"[All Fields] OR (“child, preschool"[MeSH Terms] OR (“child"[All Fields] AND “preschool"[All Fields]) OR “preschool child"[All Fields] OR “preschooler"[All Fields] OR “preschoolers"[All Fields] OR “preschool"[All Fields] OR “preschooler s"[All Fields] OR “preschools"[All Fields]) OR (“infant"[MeSH Terms] OR “infant"[All Fields] OR “infants"[All Fields] OR “infant s"[All Fields]) OR (“infant, newborn"[MeSH Terms] OR (“infant"[All Fields] AND “newborn"[All Fields]) OR “newborn infant"[All Fields] OR “newborn"[All Fields] OR “newborns"[All Fields] OR “newborn s"[All Fields]) OR (“toddler"[All Fields] OR “toddler s"[All Fields] OR “toddlers"[All Fields]) OR (“child"[MeSH Terms] OR “child"[All Fields] OR “children"[All Fields] OR “child s"[All Fields] OR “children s"[All Fields] OR “childrens"[All Fields] OR “childs"[All Fields]) OR (“adolescences"[All Fields] OR “adolescency"[All Fields] OR “adolescent"[MeSH Terms] OR “adolescent"[All Fields] OR “adolescence"[All Fields] OR “adolescents"[All Fields] OR “adolescent s"[All Fields]) OR (“adolescent"[MeSH Terms] OR “adolescent"[All Fields] OR “teen"[All Fields]) OR (“adolescent"[MeSH Terms] OR “adolescent"[All Fields] OR “youth"[All Fields] OR “youths"[All Fields] OR “youth s"[All Fields]
3	"exercise"[MeSH Terms] OR “exercise"[All Fields] OR “exercises"[All Fields] OR “exercise therapy"[MeSH Terms] OR (“exercise"[All Fields] AND “therapy"[All Fields]) OR “exercise therapy"[All Fields] OR “exercise s"[All Fields] OR “exercised"[All Fields] OR “exerciser"[All Fields] OR “exercisers"[All Fields] OR “exercising"[All Fields] OR (“exercise"[MeSH Terms] OR “exercise"[All Fields] OR (“physical"[All Fields] AND “activity"[All Fields]) OR “physical activity"[All Fields]) OR (“exercise"[MeSH Terms] OR “exercise"[All Fields] OR (“activities"[All Fields] AND “physical"[All Fields]) OR “activities physical"[All Fields]) OR (“exercise"[MeSH Terms] OR “exercise"[All Fields] OR (“activity"[All Fields] AND “physical"[All Fields]) OR “activity physical"[All Fields]) OR (“exercise"[MeSH Terms] OR “exercise"[All Fields] OR (“physical"[All Fields] AND “activities"[All Fields]) OR “physical activities"[All Fields]) OR (“exercise"[MeSH Terms] OR “exercise"[All Fields] OR (“exercise"[All Fields] AND “physical"[All Fields]) OR “exercise physical"[All Fields]) OR (“exercise"[MeSH Terms] OR “exercise"[All Fields] OR (“physical"[All Fields] AND “exercise"[All Fields]) OR “physical exercise"[All Fields]) OR (“exercise"[MeSH Terms] OR “exercise"[All Fields] OR (“physical"[All Fields] AND “exercises"[All Fields]) OR “physical exercises"[All Fields]) OR (“exercise"[MeSH Terms] OR “exercise"[All Fields] OR (“acute"[All Fields] AND “exercise"[All Fields]) OR “acute exercise"[All Fields]) OR (“exercise"[MeSH Terms] OR “exercise"[All Fields] OR (“acute"[All Fields] AND “exercises"[All Fields]) OR “acute exercises"[All Fields]) OR (“exercise"[MeSH Terms] OR “exercise"[All Fields] OR (“exercise"[All Fields] AND “acute"[All Fields]) OR “exercise acute"[All Fields]) OR (“exercise"[MeSH Terms] OR “exercise"[All Fields] OR (“exercises"[All Fields] AND “acute"[All Fields]) OR “exercises acute"[All Fields]) OR (“exercise"[MeSH Terms] OR “exercise"[All Fields] OR (“exercise"[All Fields] AND “isometric"[All Fields]) OR “exercise isometric"[All Fields]) OR (“exercise"[MeSH Terms] OR “exercise"[All Fields] OR (“exercises"[All Fields] AND “isometric"[All Fields]) OR “exercises isometric"[All Fields]) OR (“exercise"[MeSH Terms] OR “exercise"[All Fields] OR (“isometric"[All Fields] AND “exercises"[All Fields]) OR “isometric exercises"[All Fields]) OR (“exercise"[MeSH Terms] OR “exercise"[All Fields] OR (“isometric"[All Fields] AND “exercise"[All Fields]) OR “isometric exercise"[All Fields]) OR (“exercise"[MeSH Terms] OR “exercise"[All Fields] OR (“exercise"[All Fields] AND “aerobic"[All Fields]) OR “exercise aerobic"[All Fields]) OR (“exercise"[MeSH Terms] OR “exercise"[All Fields] OR (“aerobic"[All Fields] AND “exercise"[All Fields]) OR “aerobic exercise"[All Fields]) OR (“exercise"[MeSH Terms] OR “exercise"[All Fields] OR (“aerobic"[All Fields] AND “exercises"[All Fields]) OR “aerobic exercises"[All Fields]) OR (“exercise"[MeSH Terms] OR “exercise"[All Fields] OR (“exercises"[All Fields] AND “aerobic"[All Fields]) OR “exercises aerobic"[All Fields]) OR (“exercise"[MeSH Terms] OR “exercise"[All Fields] OR (“exercise"[All Fields] AND “training"[All Fields]) OR “exercise training"[All Fields]) OR (“exercise"[MeSH Terms] OR “exercise"[All Fields] OR (“exercise"[All Fields] AND “trainings"[All Fields]) OR “exercise trainings"[All Fields]) OR (“exercise"[MeSH Terms] OR “exercise"[All Fields] OR (“training"[All Fields] AND “exercise"[All Fields]) OR “training exercise"[All Fields]) OR (“exercise"[MeSH Terms] OR “exercise"[All Fields] OR (“trainings"[All Fields] AND “exercise"[All Fields]))
4	#1 and #2 and #3

### 2.2. Selection criteria

Published studies were considered to be eligible for inclusion if they met the following criteria: (a) Studies were RCTs published in English; (b) Participants included had to be children and adolescents (age <18 years) and meet the asthma diagnostic criteria in the GINA guidelines; (c) Exercise intervention should include different forms of PA in the experimental group for at least 4 weeks; (d) The outcomes of studies had to report lung function or QoL at the end.

Studies were excluded if they failed to meet the inclusion criteria. Two authors independently performed the primary literature screening. A third author was consulted for any disagreements between the two authors.

### 2.3. Data extraction

Two authors extracted data from the full text of the final included studies. Data extracted included author, year, country, participant, age, group, intervention, and outcome. In this regard, interventions and outcomes would be reported in more detail.

### 2.4. Quality assessment

The methodological quality of each included study was assessed using the Cochrane Collaboration tool by two independent authors, in which included the following seven contents: random sequence generation, allocation concealment, blinding of participants and personnel, blinding of outcome assessment, incomplete outcome data, selective reporting, and other biases. And each item was considered to high risk, low risk, or unclear. All disagreements between the two authors were in consultation with the third author.

### 2.5. Meta-analysis

In the studies we included, the control group received conventional medication therapy, while the intervention group was treated with PA in addition to the control group. This meant that we compared PA and non-PA. The meta-analysis included studies with one of the following results: the forced expiratory volume in the first second (FEV_1_), the forced vital capacity (FVC), FEV_1_/FVC, the forced expiratory flow between 25% and 75% of forced vital capacity (FEF_25–75_), or the peak expiratory flow (PEF), and the QoL assessed by the Pediatric Asthma Quality of Life Questionnaire (PAQLQ) (21). Data analysis was performed using Review Manager software version 5.3. The mean difference (MD) and the 95% confidence interval (CI) and weight between groups were calculated by the mean and standard deviation (SD) of post-intervention from groups. And due to the diversity of intervention, we used a random-effect model. A *p-*value below 0.05 was deemed statistically significant. We combined the values when two or more experimental groups were reported.

We judged heterogeneity by *χ*^2^ test, and when *p-*value was less than 0.10, it indicated statistical significance. Meanwhile, when *I^2^* was more than 50% by *I*^2^ test, it indicated moderate-to-high heterogeneity. We conducted sensitivity analyses by excluding one study sequentially and comparing the results using a random-effect model and fixed-effect model. Based on the Grading of Recommendations Assessment, Development, and Evaluation (GRADE) system, we assessed the quality of evidence in the included studies.

### 2.6. Summary of findings

We used the GRADEprofiler software to summarize the main findings of studies and to assess the certainty of the evidence, including the lung function, QoL and airway inflammation.

## 3. Results

**Figure 1:**
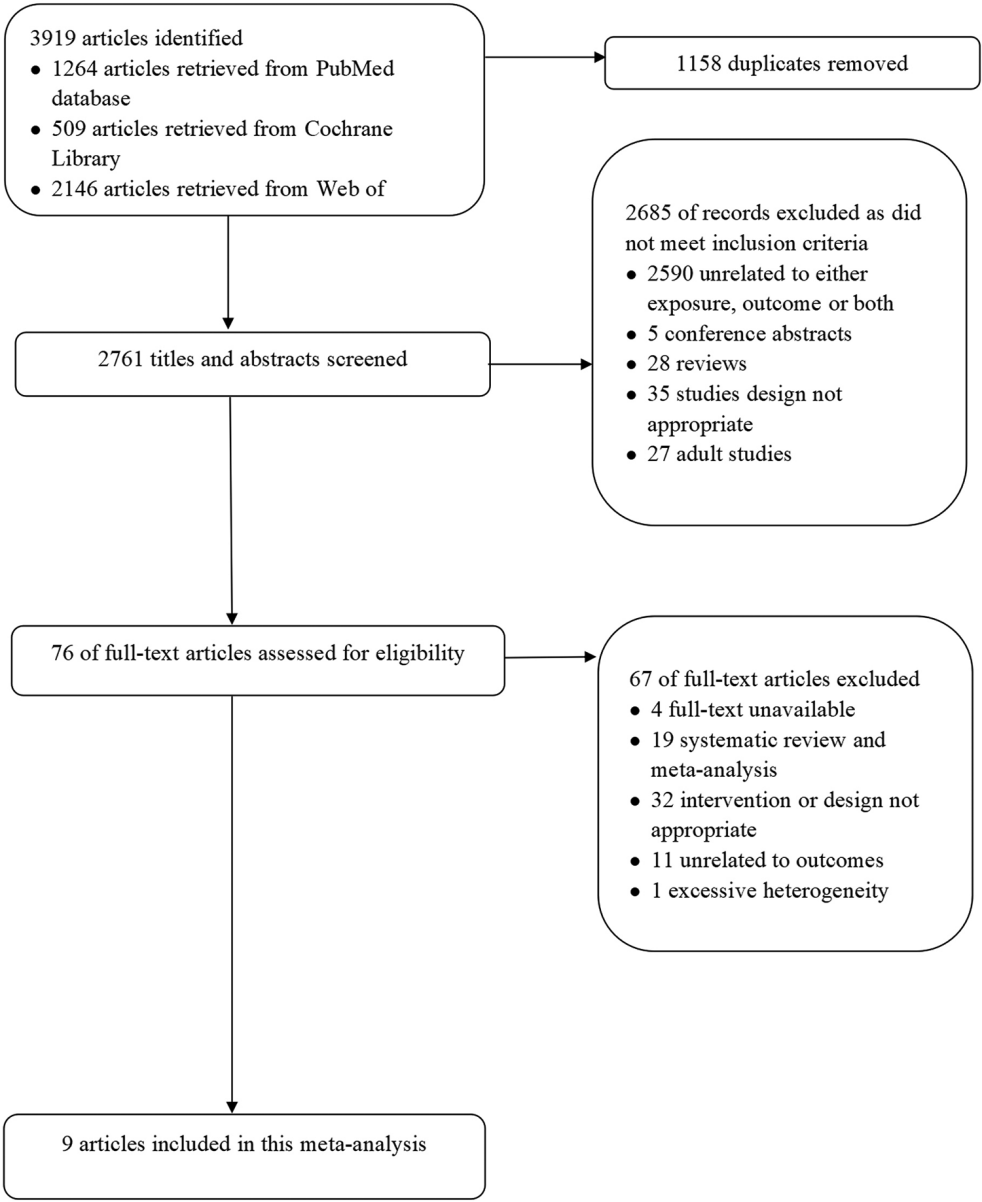
Flow diagram of studies from search to inclusion.

We identified 3,919 records through three databases, and removed 1,158 duplicates automatically and manually. There were 2,761 records remained, of which 2,685 records were excluded from screening the titles and abstracts. The remaining 76 full-text articles were assessed for eligibility, and 67 articles were excluded due to inappropriate intervention, study design, outcome, or other reasons. Finally, 9 studies (22–30) were meta-analyzed. The PRISMA flowchart illustrated this search process (Figure 1).

### 3.1. Study characteristics and types of PA

The detailed characteristics of studies were described in Table 2. A total of 496 children of 6–18 years of age were included in this meta-analysis, and interventions of all the 9 studies were different types and intensities of PA. Types of PA included high-intensity interval training (HIIT) (22), aerobic exercise on a treadmill (23, 24), riding a bicycle (25), Tai-Chi-Chuan (27), physical exercise portfolio (26, 30), swimming (28, 29), basketball (28) and football (28). The majority of PA were delivered over a time period ranging from 6 to 12 weeks. The duration of PA ranged from 30 to 60 min per section, and training frequency was from one session to three sessions per week. Five studies (22–25, 27) used heart rate (HR) to assess the exercise intensity, with the exception of the HIIT experiment that required more than 90% HR max (22), the other studies were ranging from 50% and 80% HR max (23–25, 27). The studies were conducted in Brazil, China, Denmark, Egypt, Ireland, Portugal, Spain, Turkey, and UK.

**Table 2:** Characteristics of include studies.

Study	Country	Participants	Age	Interventions	Durations	Outcomes
Abdelbasset WK 2018	Egypt etc.	Moderate persistent asthma IG: *n* = 19 CG: *n* = 19	8–12 years	Moderate-intensity aerobic exercise program [exercise training at 50%–70% of the maximum heart rate (HR max)] walking on a treadmill	10 weeks; 3 times per week; each exercise session lasting for 40 min.	Pulmonary functions (FEV_1_, FVC); aerobic capacity QoL by PAQLQ
Andrade LB 2014	Brazil	Persistent moderate asthma IG: *n* = 14 CG: *n* = 19	6–17 years	Aerobic training performed on an electric treadmill at 70%–80% of the maximum heart rate.	6 weeks; 3 times per week; each exercise session lasting for 40–50 min	Plasma cytokine; pulmonary functions (FEV_1_, FVC, FEV_1_/FVC, PEF); QoL by PAQLQ
Carew C 2018	Ireland	Mild or moderate asthma IG: *n* = 27 CG: *n* = 10	9–16 years	Swimming, football, and basketball dynamic warm-up, speed work, conditioning games/drills and cool down	6 weeks; once a week; each exercise session lasting for 40 min.	Lung function (FVC, FEV_1_, FEV_1_/FVC, PEF)
Latorre-Román PÁ 2014	Spain	Stable asthma IG: *n* = 58 CG: *n* = 47	11.53 ± 1.20 years	Low intensity to high intensity interval training Various types	12 weeks; 3 times per week; each exercise session lasting for 60 min.	QoL by PAQLQ Lung function (FEV_1_, FEV_6_, PEF)
Lin HC 2017	China	Mild asthma IG: *n* = 20 CG: *n* = 9	School children	Tai-Chi-Chuan course was specifically designed as a therapy for asthmatic children including moderate-intensity exercise with about ten minutes of higher-intensity activity	12 weeks; once a week; each exercise class for 60 min.	QoL by Standardized Pediatric Asthma Quality of Life Questionnaire; lung function (FEV_1_, FVC, FEV_1_/FVC, PEF rate); FeNO;
Onur E 2011	Turkey	Stable asthma IG: *n* = 15 CG: *n* = 15	8–13 years	Bicycle training at 50%–80% of the maximum heart rate.	8 weeks; twice per week; each exercise session lasting for 60 min.	Malondialdehyde; glutathione peroxidase; superoxide dismutase; total nitric oxide; lung function (FEV_1_, FVC)
Silva D 2013	Portugal	Controlled asthma IG: *n* = 15 CG: *n* = 15	13 ± 3 years	Exercise training include aerobic, strength, balance, and coordination exercises.	3 months; twice a week; 50 min per session.	QoL by PAQLQ
Wicher IB 2010	Denmark	Moderate persistent atopic asthma IG: *n* = 30 CG: *n* = 31	6–18 years	Different swimming session divided by skill level after warming up	3 months; twice a week; each swimming session lasted 60 min.	Lung function (FEV1, FVC, FEV_1_/FVC, FEF_25−75_); methacholine challenge test.
Winn CON 2019	UK	Stable asthma IG: *n* = 44 CG: *n* = 88	School children	High-intensity interval training; exercise activities designed to elicit a heart rate of >90% of HR maximum	6 months; 3 times per week; 30 min per sessions; 1:1 work-to-rest ratio.	Anthropometrics, Lung function (FEV_1_, FVC, FEV_1_/FVC, FEF_25−75_, PEF), FENO, Asthma control, Asthma-related quality of life, QoL by PAQLQ Cardiorespiratory fitness.

IG, intervention group; CG, control group; FEV_1_, forced expiratory volume in the first second; FVC, forced vital capacity; FEF_25−75_, forced expiratory flow between 25% and 75% of forced vital capacity; PEF, peak expiratory flow; QoL, quality of life; PAQLQ, the Pediatric Asthma Quality of Life Questionnaire; FEV_6_, forced expiratory volume in sixth second;. PC20, provocative concentration of methacholine causing a 20% fall in FEV_1_; FeNO, fractional exhaled nitric oxide.

### 3.2. Risk of bias in the included studies

**Figure 2:**
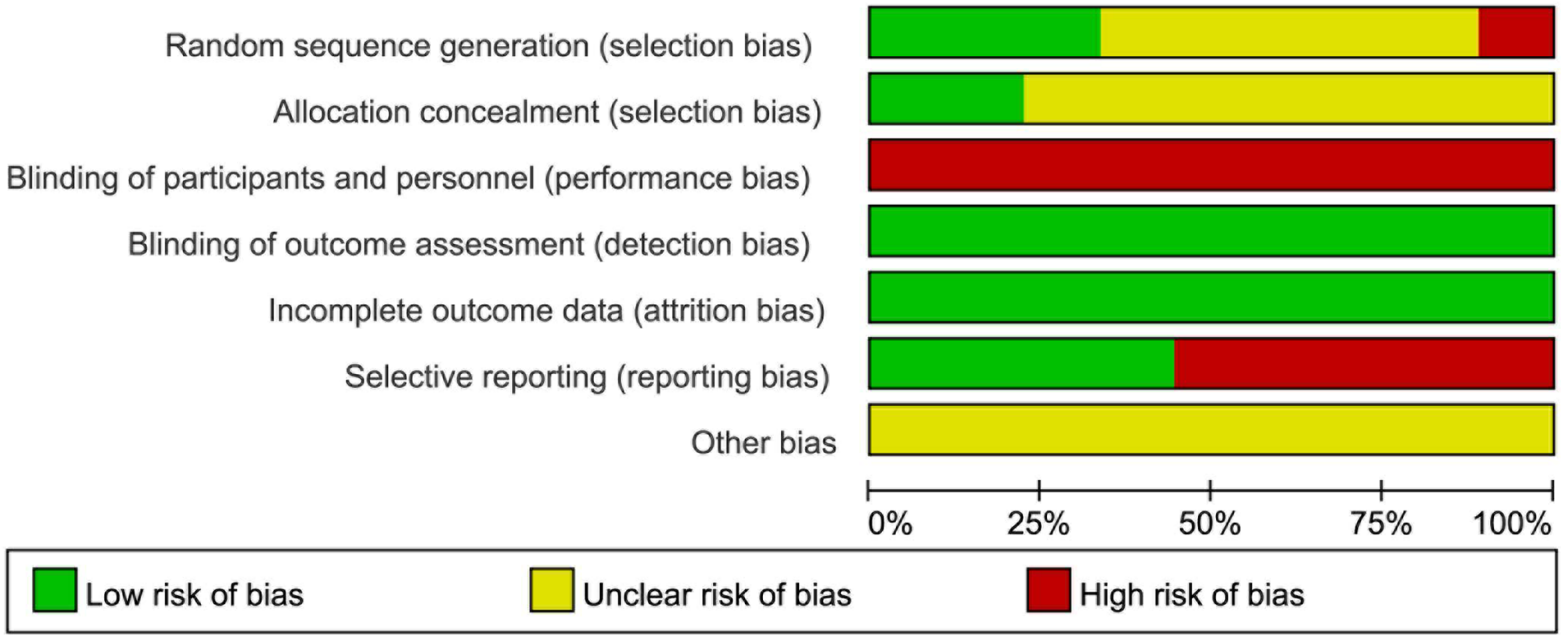
Risk of bias graph.

**Figure 3:**
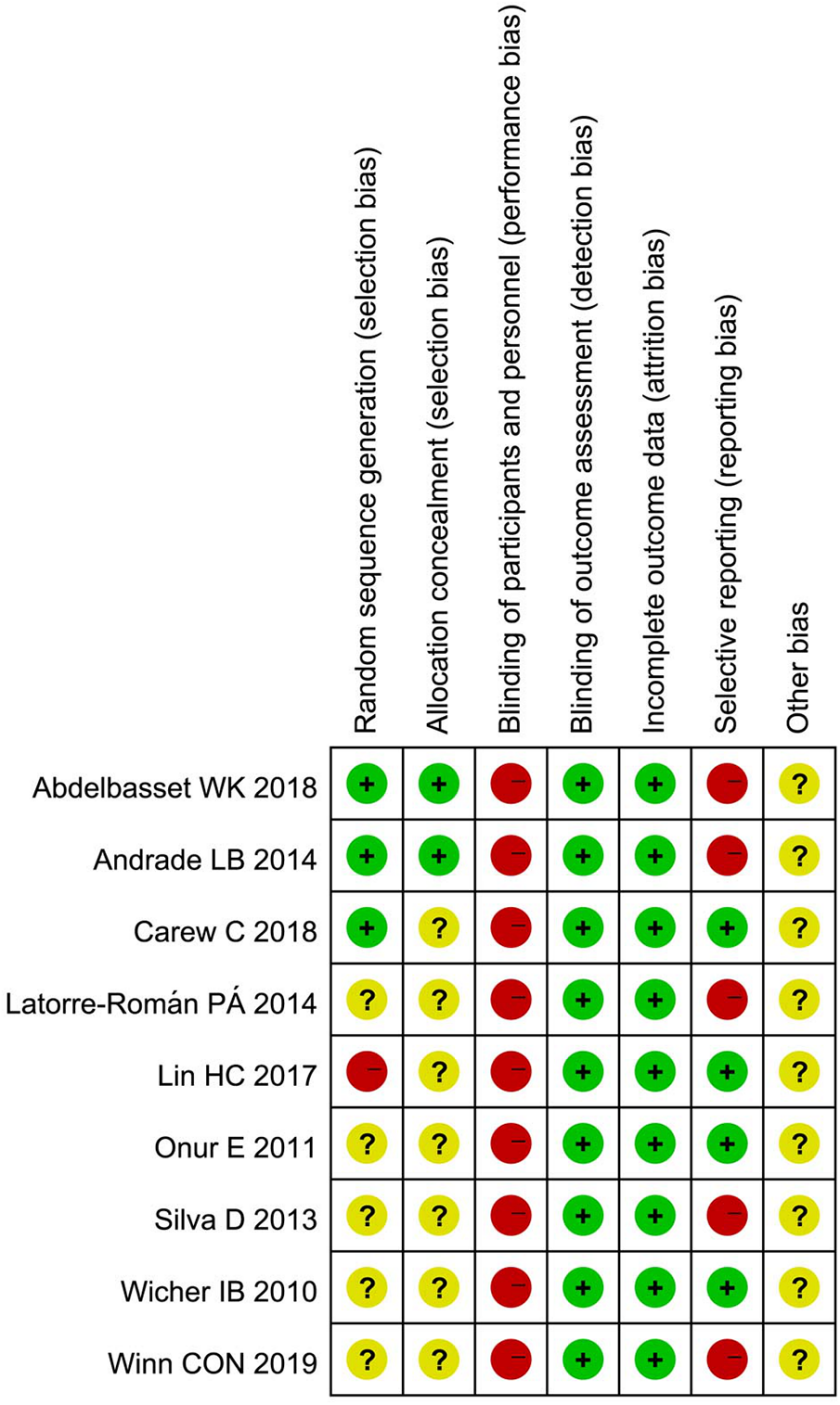
Risk of bias summary.

We summarized the risk of bias for each included study in Figure 2, and presented each item as a percentage in Figure 3.

#### 3.2.1. Random sequence generation

Eight of included studies (22–26, 28–30) were randomly allocated, in which only three studies described the details of randomized method and thus were assessed as low risk of bias (22, 24, 28). One study (27) was allocated based on the participants' intention and was judged as high risk of bias. The remaining five studies were classified as unclear risk of bias due to a lack of random sequence generation (23, 25, 26, 29, 30).

#### 3.2.2. Allocation concealment

The allocation list of two studies was opened after the groups were formed so they were assessed as low risk of bias (23, 24). The other seven studies were deemed to be unclear risk of bias for allocation concealment (22, 25–30).

#### 3.2.3. Blinding

Since interventions involved PA and participants were required to perform it, blinding was not possible and all the studies were considered high risk.

#### 3.2.4. Incomplete outcome data

All studies with a withdrawal were assessed as low risk, because the missing data was unlikely to influence the true outcome.

#### 3.2.5. Selective reporting

Since the assessment of QoL was based on subjective judgment, there were risks in reporting. And five studies (22–24, 26, 30) that reported QoL were deemed to be high risk and the remaining were judged to be low risk.

#### 3.2.6. Other bias

Because of the insufficient evidence, all studies were assessed as unclear risk.

### 3.3. Effects of interventions

Effects of PA intervention on lung function, QoL and airway inflammation were shown in Table 3. We downgraded some of the evidence and justified it.

**Table 3:** Summary of findings.

PA for lung function						
**Patient or population:** children with asthma **Settings:** outpatient **Intervention:** PA						
**Outcomes**	Illustrative comparative risks* (95% CI)		**Relative effect** **(95% CI)**	**No of Participants** **(studies)**	**Quality of the evidence** **(GRADE)**	**Comments**
	Assumed risk	Corresponding risk				
** **	**Control**	**Lung function**	** **	** **	** **	** **
**lung function - FEV_1_**		The mean lung function - FEV_1_ in the intervention groups was **3.86 higher** (1.15 to 6.58 higher)		292 (6 studies)	⊕⊕⊕⊝ **moderate**^1^	MD 3.17 (−2.82 to 9.15)
**lung function - FVC**		The mean lung function - FVC in the intervention groups was **8.16 higher** (5.1 to 11.21 higher)		292 (6 studies)	⊕⊕⊕⊝ **moderate**^1^	MD 7.62 (3.46 to 11.78)
**lung function - FEV_1_/FVC**		The mean lung function - FEV_1_/FVC in the intervention groups was **0.84 higher** (1.51 lower to 3.2 higher)		224 (4 studies)	⊕⊕⊕⊝ **moderate**^1^	MD 0.73 (−2.76 to 4.22)
**lung function - FEF_25__−75_**		The mean lung function - FEF_25__−75_ in the intervention groups was **10.39 higher** (2.96 to 17.82 higher)		158 (2 studies)	⊕⊕⊕⊝ **moderate**^1^	MD 10.39 (2.96 to 17.82)
**lung function - PEF**		The mean lung function - PEF in the intervention groups was **0.44 higher** (6.71 lower to 7.58 higher)		134 (2 studies)	⊕⊕⊕⊝ **moderate**^1^	MD 0.44 (−6.71 to 7.58)
*The basis for the **assumed risk** (e.g. the median control group risk across studies) is provided in footnotes. The **corresponding risk** (and its 95% confidence interval) is based on the assumed risk in the comparison group and the **relative effect** of the intervention (and its 95% CI). **PA:** Physical activity; **CI:** Confidence interval; **FEV_1_:** forced expiratory volume in the first second; **FVC:** forced vital capacity; **FEF_25_****_−75_:** forced expiratory flow between 25% and 75% of forced vital capacity; **PEF:** peak expiratory flow.						
GRADE Working Group grades of evidence **High quality:** Further research is very unlikely to change our confidence in the estimate of effect. **Moderate quality:** Further research is likely to have an important impact on our confidence in the estimate of effect and may change the estimate. **Low quality:** Further research is very likely to have an important impact on our confidence in the estimate of effect and is likely to change the estimate. **Very low quality:** We are very uncertain about the estimate.						
^1^ not fully blind						
**PA for QoL**						
**Patient or population:** children with asthma **Settings:** outpatient **Intervention:** PA						
**Outcomes**	Illustrative comparative risks* (95% CI)		**Relative effect** **(95% CI)**	**No of Participants** **(studies)**	**Quality of the evidence** **(GRADE)**	**Comments**
	Assumed risk	Corresponding risk				
** **	**Control**	**QoL**	** **	** **	** **	** **
**PAQLQ - Overall**		The mean PAQLQ - overall in the intervention groups was **1.38 standard deviations higher** (0.26 to 2.5 higher)		298 (5 studies)	⊕⊕⊝⊝ **low**^1,2^	SMD 1.38 (0.26 to 2.5)
**PAQLQ - Symptoms**		The mean PAQLQ - symptoms in the intervention groups was **1.4 standard deviations higher** (0.22 to 2.57 higher)		298 (5 studies)	⊕⊕⊝⊝ **low**^1,2^	SMD 1.4 (0.22 to 2.57)
**PAQLQ - Activity limitation**		The mean PAQLQ - activity limitation in the intervention groups was **1.37 standard deviations higher** (0.18 to 2.56 higher)		298 (5 studies)	⊕⊕⊝⊝ **low**^1,2^	SMD 1.37 (0.18 to 2.56)
**PAQLQ - Emotions function**		The mean PAQLQ - emotions function in the intervention groups was **1.35 standard deviations higher** (0.34 to 2.36 higher)		298 (5 studies)	⊕⊕⊝⊝ **low**^1,2^	SMD 1.35 (0.34 to 2.36)
*The basis for the **assumed risk** (e.g. the median control group risk across studies) is provided in footnotes. The **corresponding risk** (and its 95% confidence interval) is based on the assumed risk in the comparison group and the **relative effect** of the intervention (and its 95% CI). **PA:** Physical activity; **QoL:** quality of life; **CI:** Confidence interval; **PAQLQ:** the Pediatric Asthma Quality of Life Questionnaire						
GRADE Working Group grades of evidence **High quality:** Further research is very unlikely to change our confidence in the estimate of effect. **Moderate quality:** Further research is likely to have an important impact on our confidence in the estimate of effect and may change the estimate. **Low quality:** Further research is very likely to have an important impact on our confidence in the estimate of effect and is likely to change the estimate. **Very low quality:** We are very uncertain about the estimate.						
^1^ not fully blind ^2^ subjective parameter						
**PA for FeNO**						
**Patient or population:** children with asthma **Settings:** outpatient **Intervention:** PA						
**Outcomes**	Illustrative comparative risks* (95% CI)		**Relative effect** **(95% CI)**	**No of Participants** **(studies)**	**Quality of the evidence** **(GRADE)**	**Comments**
	Assumed risk	Corresponding risk				
** **	**Control**	**FeNO**	** **	** **	** **	** **
**FeNO**		The mean FeNO in the intervention groups was **1.74 lower** (11.36 lower to 7.88 higher)		128 (2 studies)	⊕⊕⊕⊝ **moderate**^1^	MD −1.74 (−11.36 to 7.88)
*The basis for the **assumed risk** (e.g. the median control group risk across studies) is provided in footnotes. The **corresponding risk** (and its 95% confidence interval) is based on the assumed risk in the comparison group and the **relative effect** of the intervention (and its 95% CI). **PA:** Physical activity; **FeNO:** Fractional exhaled nitric oxide; **CI:** Confidence interval;						
GRADE Working Group grades of evidence **High quality:** Further research is very unlikely to change our confidence in the estimate of effect. **Moderate quality:** Further research is likely to have an important impact on our confidence in the estimate of effect and may change the estimate. **Low quality:** Further research is very likely to have an important impact on our confidence in the estimate of effect and is likely to change the estimate. **Very low quality:** We are very uncertain about the estimate.						
^1^ not fully blind						

### 3.4. Outcome measures and findings

Each included study compared the effects of PA in the experimental group with non-PA in the control group. There were nine studies included in the qualitative synthesis, in which outcomes of eight studies assessed lung functions, including FEV_1_ (22–29), FVC (22–25, 27–29), FEV_1_/FVC (22, 24, 27–29), PEF (22, 24, 26, 28), FEF_25–75_ (22, 29), and outcomes of six studies assessed QoL (22–24, 26, 30).

#### 3.4.1. Lung function

##### 3.4.1.1. FEV_1_

**Figure 4:**
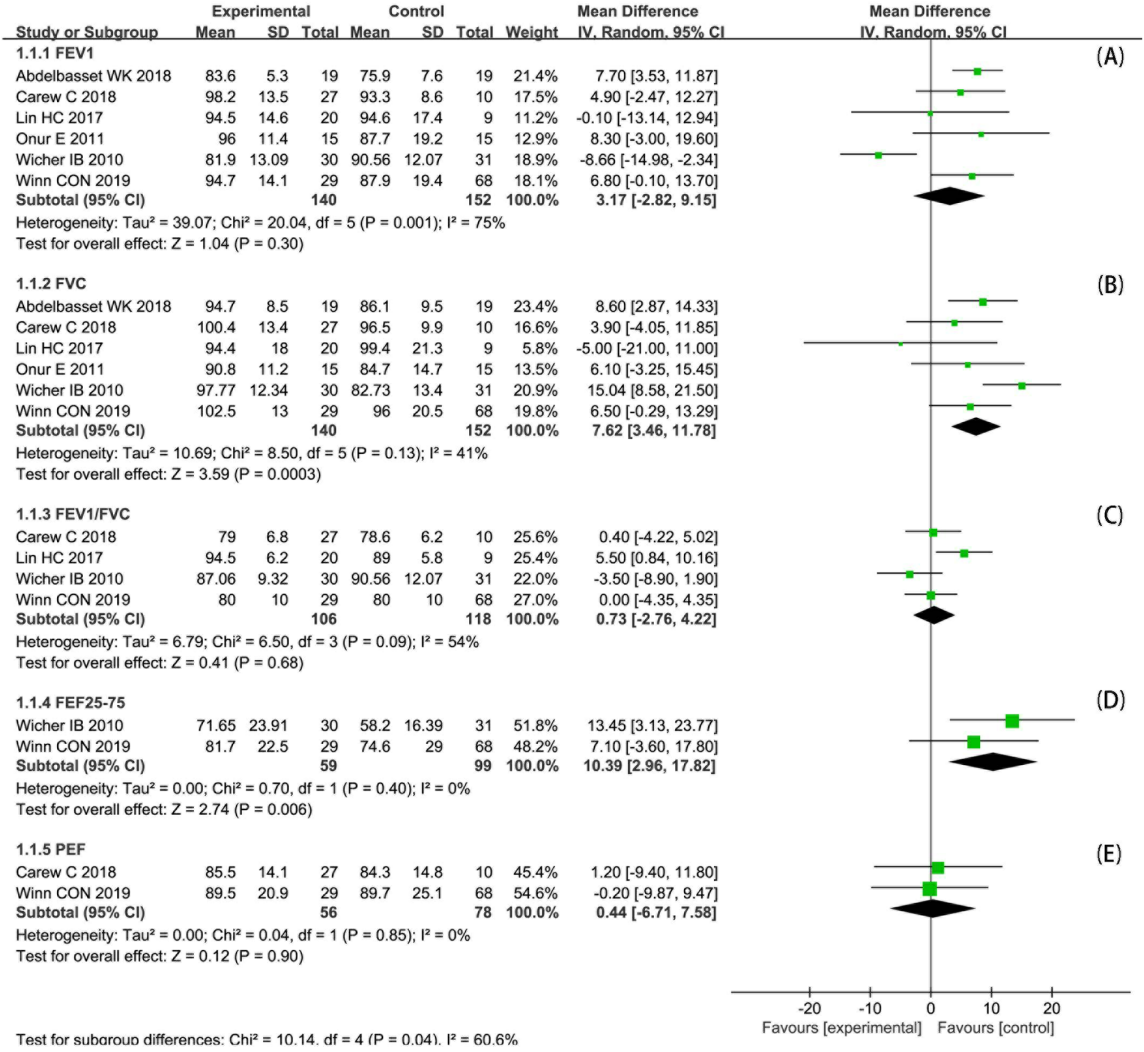
Forest plot: changes in lung function after PA.

Eight studies (22–29) assessed FEV_1_, in which significant improvements reported in four of them (25–27, 29). Since two studies (24, 26) reported only changes before and after the intervention, six studies (22, 23, 25, 27–29) were included in the meta-analysis (Figure 4A). The meta-analysis of FEV_1_%pred (FEV_1_ in percent predicted values, similarly hereinafter) included 292 participants. There was no statistically significant difference in FEV_1_%pred of post-intervention between two groups (MD 3.17; 95% CI: −2.82 to 9.15; *p* = 0.30). And the heterogeneity was high (*I*^2^ = 75%; *p* = 0.001).

##### 3.4.1.2. FVC

FVC was measured in seven studies (22–25, 27–29), in which three studies reported a significant improvement (25, 28, 29). One study (24) reported changes before and after the intervention only, so six studies (22, 23, 25, 27–29) were included in the meta-analysis (Figure 4B). The meta-analysis of FVC%pred included 292 participants. There was a statistically significant difference in FVC%pred of post-intervention between two groups (MD 7.62; 95% CI: 3.46 to 11.78; *p* < 0.001). And the heterogeneity was acceptable (*I*^2 ^= 41%; *p* = 0.13).

##### 3.4.1.3. FEV_1_/FVC

FEV_1_/FVC was assessed in five studies (22, 24, 27–29), in which one study reported a significant improvement (27). Since one study (24) reported changes before and after the intervention only, four studies (22, 27–29) were included in the meta-analysis (Figure 4C). The meta-analysis of FEV_1_/FVC included 224 participants. There was no statistically significant difference in FEV_1_/FVC of post-intervention between two groups (MD 0.73; 95% CI: −2.76 to 4.22; *p* = 0.68). And the heterogeneity was moderate (*I*^2^ = 54%; *p* = 0.09).

##### 3.4.1.4. FEF_25–75_

FEF_25–75_ was reported in two studies (22, 29), and one (22) of which reported a significant improvement. We included both studies in the meta-analysis (Figure 4D). The meta-analysis of FEF_25–75_%pred included 158 participants. There was a statistically significant difference in FEF_25–75_%pred of post-intervention between two groups (MD 10.39; 95% CI: 2.96 to 17.82; *p* = 0.006). And the heterogeneity was acceptable (*I*^2^ = 0%; *p* = 0.40).

##### 3.4.1.5. PEF

PEF was reported in four studies (22, 24, 26, 28), two (24, 26) of which reported significant improvement. Because two studies (24, 26) reported PEF that did not meet the criteria for pooling, the remaining two studies (22, 28) were included in the meta-analysis (Figure 4E). The meta-analysis of PEF%pred included 134 participants. There was no statistically significant difference in PEF%pred of post-intervention between two groups (MD 0.44; 95% CI: −6.71 to 7.58; *p* = 0.90). And the heterogeneity was acceptable (*I*^2^ = 0%; *p* = 0.85).

#### 3.4.2. Quality of life

**Figure 5:**
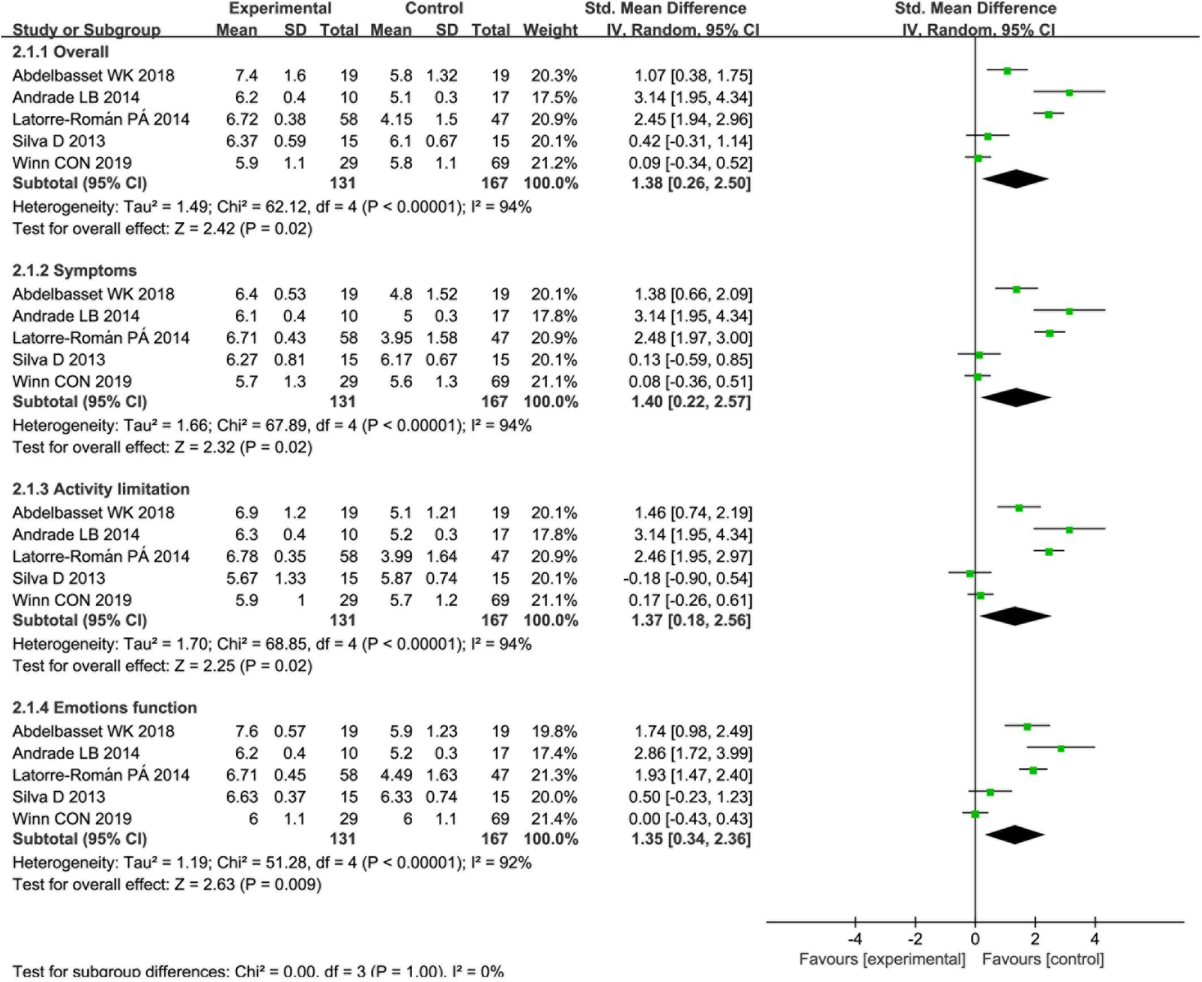
Forest plot: changes in QoL after PA.

QoL was assessed in six studies (22–24, 26, 27, 30). Five studies used the PAQLQ (22–24, 26, 30), and the remaining one study used different questionnaire (27). Meanwhile, five studies showed statistically significant improvement (22–24, 26, 27, 30), and one study had no significant statistical improvement (22). We conducted a meta-analysis of five studies that used the PAQLQ (Figure 5). The meta-analysis of QoL included 298 participants. And the meta-analysis showed that overall PAQLQ score and the three domains (symptoms, activity limitation, and emotional function) were statistically significant difference between two groups. Separately, they were overall PAQLQ score (MD 1.38; 95% CI: 0.26 to 2.50; *p* = 0.02), symptoms (MD 1.40; 95% CI: 0.22 to 2.57; *p* = 0.02), activity limitation (MD 1.37; 95% CI: 0.18 to 2.56; *p* = 0.02), and emotional function (MD 1.35; 95% CI: 0.34 to 2.36; *p* = 0.009). And all heterogeneity exceeded acceptable (*I*^2^ > 90%).

#### 3.4.3. Airway inflammation

**Figure 6:**
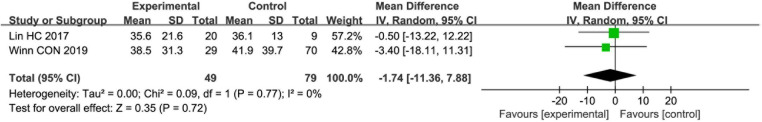
Forest plot: changes in FeNO after PA.

Fractional exhaled nitric oxide (FeNO) is the most commonly used marker of airway inflammation in asthmatic children. Two studies (22, 27) reported changes in FeNO after PA intervention, both of which decreased from baseline levels. We included both studies in the meta-analysis (Figure 6). There was no statistically significant difference in FeNO of post-intervention between two groups (MD −1.74; 95% CI: −11.36 to 7.88; *p* = 0.72). And the heterogeneity was acceptable (*I*^2^ = 0%; *p* = 0.77).

## 4. Discussion

The aim of this systematic review and meta-analysis was to synthesize the available studies investigating the effects of PA in asthmatic children.

Compared with the control groups, our results showed a significant improvement in FVC and FEF_25–75_ on lung function in experiment groups with PA. Earlier studies found that PA improved lung function in healthy children and adolescents by promoting longitudinal growth in lung volume, increasing FEV_1_ and FVC (31, 32). An RCT study showed that combined exercise training improved lung function in FEV_1_, FVC, FEF_25–75_ (33). However, in another RCT study, which was not included in the meta-analysis because of high heterogeneity, it was concluded that PA did not improve lung function (FEV_1_, FEV_1_/FVC) in children with asthma, but it did improve the clinical symptoms and QoL (34). Thus, current studies remained controversial on whether PA improved FEV_1_, but the evidence was more robust on PA improving FVC. Our study further reinforced this conclusion. Also, we found an increase in FEF_25–75_ after the intervention of PA, which suggested that the benefits of PA for asthmatic children were not only restricted to central airways but also small airways. This will require our focus in the future. Because asthmatic children who had small airway dysfunction were often poorly controlled and more likely to have frequent exacerbations (35). If PA is shown to improve the small airway dysfunction, this may provide an additional treatment option for these children. But more evidence is needed to support.

However, the specific mechanism by which PA improves lung function is still not well understood. A review concluded that the effect of exercise on lung function may be due to the ability of aerobic exercise to accelerate respiratory rate and strengthen respiratory muscles, thus contributing to the stretching of airway smooth muscle and sustained bronchial dilation (36). Onur E et al. suggested that a potential mechanism for the improvement in lung function in asthmatic children after PA might be associated with an increased oxidative capacity, leading to a reduction in oxidative burden and enhancing the anti-inflammatory effects of steroids (25).

Our review also showed significant improvement in the QoL of children with PA and was consistent with the findings of previous studies (36, 37), but we found high heterogeneity in the meta-analysis of QoL. This might be explained by the fact that the QoL couldn't be evaluated by objective indicators, and subjective judgments were mainly made by means of questionnaires. Participants were susceptible to a variety of factors that led to certain tendencies. A recent review concluded that aerobic exercise could reduce the prevalence and frequency of symptoms in children with nocturnal asthma (38). Therefore, based on the available evidence, we believed that PA could improve the quality of life of asthmatic children.

In this study, we found there was no statistically significant improvement in FeNO. This might be due to the fact that we included too few studies and that the interventions in these studies were not long enough. However, some reviews concluded that exercise could reduce levels of inflammation-related factors and cells, such as FeNO, C-reactive protein, and blood eosinophils (37, 39). Similarly, one study suggested that acute moderate-intensity exercise was associated with decreased exhaled nitric oxide (40). Also, an earlier review described in detail the possible mechanisms of the anti-inflammatory effects of exercise, which believed that anti-inflammatory effects of PA were mainly mediated by the reduction in release of adipokines and the induction of an anti-inflammatory environment through exercise (41). And other studies found that PA also increased the number of regulatory T cells, elevated the expression of anti-inflammatory cytokines, reduced the release of pro-inflammatory cytokines, and decreased the levels of pro-inflammatory cells in the blood (42, 43). These were also demonstrated in animal experiments (44–46). Therefore, we believed that PA could improve airway inflammation in asthmatic children to some extent, but higher quality clinical studies were needed.

The participants included in this study were all mild to moderate asthmatic children. Previous studies discussed the safety of PA in children with mild to moderate asthma and concluded that it was well tolerated and had a low incidence of adverse events (19). It was also concluded that the benefits of regular exercise for asthma patients far outweigh the risks (36). Therefore, we believed that PA was beneficial for children with mild to moderate asthma. In addition, a study suggested an association between poor asthma control and inadequate PA in urban children (47), and another study showed that low levels of PA led to an increased risk of new-onset asthma in children and adolescents (48). Consequently, after assessing the children's condition, physicians should advise them to exercise as much as possible according to an appropriate exercise prescription (49). We noted that some recent studies found improvement in asthma symptoms and quality of life with PA in adults with severe asthma (50, 51), but there were no studies on children yet because of the possible ethical risks.

## 5. Limitation

Our study also had some limitations. First, the exercise patterns in the study belong to moderate-to-severe intensity exercise, but there was some heterogeneity with frequency, intensity, time and type of exercise. Second, most of the interventions in the studies we included were about 12 weeks in duration, and it was not possible to assess the long-term effects of exercise on patients. Third, we did not make comparisons between different PA, and this prevented us from determining which PA was most beneficial for asthmatic children. One of the studies we included concluded that swimming might have greater benefits for asthmatic children than other forms of exercise (28). Four, although the inclusion of earlier studies might increase heterogeneity, their exclusion would also introduce some bias. Finally, the comprehensiveness of the results in the review might be hindered by the fact that this review only collected three of the most commonly used databases and was limited to studies published in English.

## 6. Conclusion

In summary, our systematic review and meta-analysis suggests that PA can improve FVC, FEF_25–75_, and quality of life in asthmatic children, while more evidence is needed for its effect on lung function. And there is insufficient clinical evidence of improvement in airway inflammation. However, we believe PA as an adjunctive therapy should be recommended for asthmatic children, except for severe asthma or exacerbations. In the future, more studies are still needed to explore the specific mechanisms of PA in improving lung function and airway inflammation. Also, it is worthwhile to continue research on what type of PA is more beneficial for asthma.

## Data Availability

The original contributions presented in the study are included in the article/Supplementary Material, further inquiries can be directed to the corresponding author.
